# Branching architecture limits the number of fixed somatic mutations in trees

**DOI:** 10.1093/g3journal/jkaf234

**Published:** 2025-12-10

**Authors:** Frank Johannes

**Affiliations:** Plant Epigenomics, Technical University of Munich, Emil-Ramann-Str. 4, Freising 85354, Germany

**Keywords:** somatic mutations, somatic epimutations, mutation rate, epimutation rate, life-history traits, branching, plant development

## Abstract

Trees are long-lived plants characterized by the development of highly branched shoot systems. Along these structures, somatic mutations arise and may become fixed in reproductive tissues such as flowers and fruits. Because mature trees produce tens of thousands of terminal branches, limiting the accumulation of somatic mutations is critical to avoid mutational meltdown and inbreeding depression. Although recent evidence suggests that long-lived plants have evolved mechanisms that slow the buildup of somatic variants with age, the developmental basis for this remains unclear. Here, we derive a theoretical model linking crown development with cell lineage sampling to show that branching architecture strongly influences the accumulation of unique somatic mutations, often to the same extent as modulating the mutation rate itself. We find that tree forms that promote developmental path-sharing among branches restrict the spread of distinct cell lineages, lowering the crown-wide mutation burden by orders of magnitude even when mutation rates and branch numbers are held constant. This buffering effect suggests that branching strategies may evolve not only to optimize growth and resource allocation, but also to limit the genomic variation generated during ontogeny.

## Introduction

**Fig. 1.**
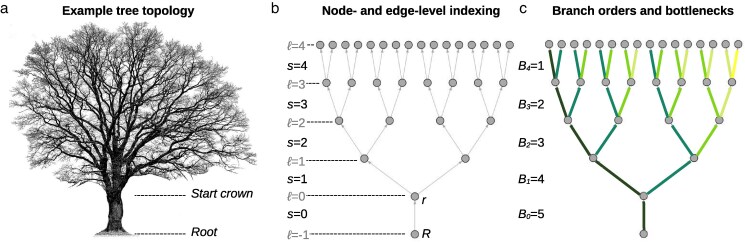
Graph representation of the tree crown. a) Trees develop complex, highly branched shoot systems as they grow. An example of an oak tree is shown. Mature specimens can sustain hundreds of thousands of terminal branches. b) The branching structure of a tree can be represented as a connected, acyclic graph with edges corresponding to internode segments and nodes to branch points. Node *R* denotes the actual root of the tree and node *r* marks the start of the tree crown. The crown itself is modeled here as a perfectly balanced binary tree, in which each internal node has two children. The hierarchical structure of the tree is quantified by its depth *D* (here D=4), the maximum distance from the crown base to the terminal nodes. Node levels are indexed as ℓ∈{−1,0,…,D} and edge (segment) levels as s∈{0,…,D}. c) For real trees, it is useful to assign each segment a *branch order* so that the main axis can be distinguished from its lateral derivatives. Knowing the order allows one to count, for any segment at level *s*, how many laterals branch off above it. This count is exactly the number of cellular bottlenecks a mutation arising on that segment must pass to reach a terminal tip and is given by $B_{s}=D-s$ for the balanced case illustrated.

Trees are long-lived organisms that gradually develop complex, highly branched shoot systems over time (Fig. 1a). In many species, mature individuals can sustain tens of thousands of terminal branches, each derived from a shoot apical meristem (SAM), a small, self-renewing population of stem cells located at the shoot apex. Because the SAM gives rise to the entire shoot system, somatic mutations arising in this region can be perpetuated across large sectors of the plant body (Iwasa et al. 2023; Tomimoto and Satake 2023; Chen et al. 2024; Johannes 2025). These mutations may accumulate over time and be retained in both vegetative structures (e.g. leaves, stems) and reproductive tissues (e.g. seeds, fruits). As a result, they can influence not only the development and physiology of the individual tree, but also its genetic contribution to the next generation (Schoen and Schultz 2019; Quiroz et al. 2023).

Plants with long life spans appear to have evolved strategies to mitigate the detrimental effects of mutation load and reduce the risk of inbreeding depression. These include low selfing rates and large effective population sizes, both of which help maintain genetic diversity and minimize inbreeding coefficients (Scofield and Schultz 2006; Schoen and Schultz 2019; Lesaffre 2021). In addition to these population-level adaptations, emerging evidence points to intrinsic mechanisms that actively slow the accumulation of somatic mutations per unit time (Lanfear et al. 2013). This is apparent in the reduced somatic mutation rate seen in long-lived tree species like *Osmanthus fragrans*, which is about two orders of magnitude lower per year than in shorter-lived species like *Salix suchowensis* (Johannes 2024; De La Torre et al. 2017). A similar trend has been observed at the epigenetic level, where epimutation rates, measured as stochastic gains and losses of DNA methylation, are negatively correlated with generation time. This parallel reduction suggests a shared underlying biological mechanism that affects both genetic and epigenetic mutation processes (Johannes 2024). However, the exact nature of this mechanism remains unknown. One hypothesis is that long-lived trees simply slow meristematic cell divisions per unit time (Lanfear et al. 2013). This mechanism would reduce errors arising from DNA synthesis and DNA methylation maintenance during replication. Although direct *in vivo* validation of this hypothesis remains challenging, it is strongly supported by the recent observation that somatic (epi)mutation rates correlate positively with growth rates and cell division frequency in vegetative tissues (Lanfear et al. 2013; Zhou et al. 2025).

The link between growth dynamics and somatic mutation accumulation suggests a broader connection between molecular evolution and life-history traits. Since SAM-derived mutations are primarily incorporated into lateral organs during branch initiation, the spatial and temporal dynamics of branching may present a major rate-limiting step for the accumulation of mutations in the tree crown, a possibility that has received little attention. Here, we present a theoretical analysis to investigate this relationship. We demonstrate that branching architectures and branch age distributions can substantially impact the accumulation of new somatic mutations, with effects comparable to modulating the mutation rate itself. Tree structures that maximize developmental path sharing, where many terminal branches trace back to a common meristematic lineage, tend to suppress mutational diversity in the crown. In some cases, these architectural features can reduce the mutation burden by orders of magnitude, even when baseline mutation rates and terminal branch numbers remain unchanged.

Our findings have major implications for understanding the rate and topological distribution of somatic mutations in trees, and highlight life-history traits as key determinants of intra-organism somatic heterogeneity.

## Theory and discussion

### Modeling the spatio-temporal architecture of a tree crown

#### A graph representation of the crown.

We model the tree crown as a rooted, directed acyclic graph A=(V,E), in which each vertex (*V*) represents a branch point and each directed edge (*E*) represents an internodal segment (Fig. 1a–c, Table 1). A special root node *R* connects the basal segment (s=0) to the first crown branch point *r* at level 0. Above this, we assume that the crown forms a perfectly balanced binary structure of depth *D*, with level ℓ containing $2^{\ell }$ vertices (ℓ=0,1,…,D). Each vertex at level s−1 gives rise to two child segments at level *s*, so that every developmental path from *R* to a terminal meristem consists of exactly D+1 segments indexed by s=0,1,…,D.

**Table 1.** Key notation and indexing used in the tree-crown model.

Symbol	Meaning
*D*	Crown depth (number of bifurcation levels)
*s*	Segment level, s=0,…,D
$2^{s}$	Number of internode segments at level *s*
$B_{s}$	Branch points above level *s*, $B_{s}=D-s$
*T*	Total developmental time per root-to-tip path
*λ*	Exponential tilt parameter for time allocation
$w_{s}(\lambda )$	Proportion of *T* allocated to level *s*
$t_{s}$	Time on level *s*, $t_{s}=T\;w_{s}(\lambda )$
$p_{\det}(s)$	Detection probability, $1-(1-1/m{)}^{B_{s}+1}$
A(λ)	Architectural factor, $\sum_{s=0}^{D}2^{s}w_{s}(\lambda )p_{\det}(s)$

At crown level *s*, there are $2^{s}$ internode segments, each representing a distinct developmental lineage. The number of branch points that remain above a segment at that level is


$$B_{s}=D-s,$$

which defines the number of potential bottlenecks a mutation on segment *s* must pass through to reach a terminal meristem. When analyzing real trees, the counting of branch points requires that each segment is assigned a *branch order*. The basal segment has order zero; at each branching point, the continuation segment retains the parent’s order, while the lateral segment increases its order by one. This indexing scheme distinguishes the main axes from lateral derivatives and determines how many lateral branches arise from a given axis (Fig. 1c). Together, these structural quantities provide the topological foundation for all subsequent derivations of the crown-wide mutation burden.

#### Developmental time allocation.

**Fig. 2.**
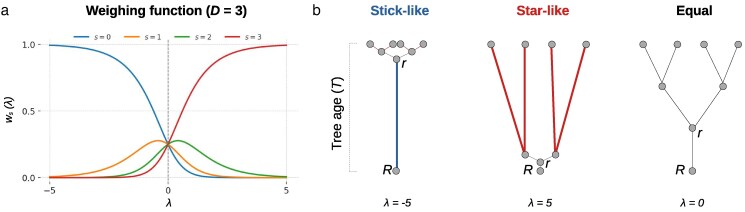
Developmental time allocation. a) Exponential tilt function $w_{s}(\lambda )$ allocates time $t_{s}$ to internode segments *s* (colored lines). For D=3, the plot shows how the parameter *λ* governs time allocation. For large negative *λ*, developmental time is allocated to the root segment s=0 (blue line); for large positive *λ* time is shifted toward the terminal segment s=3 (red line) and for λ=0, time is allocated equally across segments (intersection points of lines). b) Topological consequences of the time allocation regimes described in A.

Having established the basic tree topology, let us now use *T* to denote the total developmental time from root *R* to tip *D*. It can be measured in terms of years, number of mitotic cell divisions or any other unit of time. We partition *T* across the D+1 internode segments by introducing an exponential tilt function. Define


$$w_{s}(\lambda )=\frac{e^{\lambda s}}{\sum_{r=0}^{D}e^{\lambda r}},\;t_{s}=T\;w_{s}(\lambda ),$$

so that $\sum_{s}w_{s}(\lambda )=1$ and $\sum_{s}t_{s}=T$. Here, $t_{s}$ is the time assigned to internode segment *s*. By modulating the parameter λ, this tilt function allows us to explore diverse growth regimes. For example, in the limiting case where λ→−∞, we have that


$$w_{s}(\lambda )\to \left\{ \begin{array}{cc} 1, & s=0,\\ 0, & s>0, \end{array}\right.$$

meaning that all time is concentrated on the basal segment. This growth regime produces a “stick-like” tree architecture (Fig. 2b, left panel). When λ=0, $w_{s}=1/(D+1)$ for every *s*, yielding uniform time allocation (Fig. 2b, right panel). At the other extreme, λ→+∞,


$$w_{s}(\lambda )\to \left\{ \begin{array}{cc} 1, & s=D,\\ 0, & s<D, \end{array}\right.$$

so that all time is allocated to the terminal segment. This growth regime yields a “star-like” tree architecture (Fig. 2b, middle panel). Hence, by tuning *λ*, this one-parameter family smoothly shifts developmental strategies from trunk-dominated through uniform to tip-dominated growth.

### Mutation sampling and propagation

**Fig. 3.**
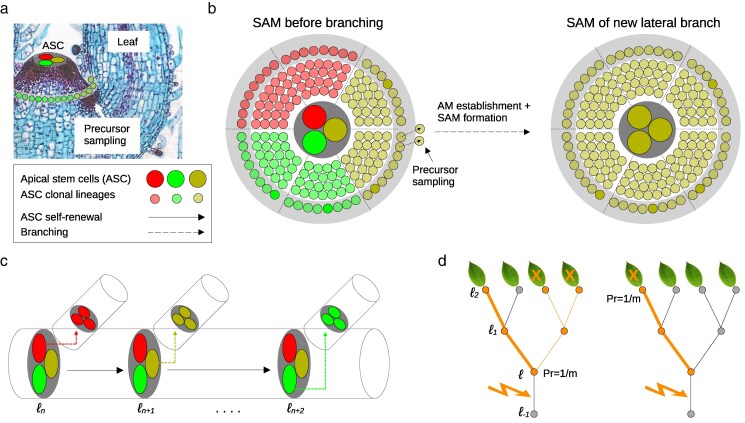
Branching model and ASC sampling. a) Side-view of the SAM. The colored circles at the tip represent three (m=3) ASC. Clonal descendants of these ASCs are shown in corresponding colors at the SAM periphery. The arrow points to the site of AM establishment. b) A schematic top-view of the SAM. The tree clonal sectors are shown just before branching, with each sector corresponding to one of the ASC at the tip. During branching, precursors are “sampled” from the SAM periphery to acts as founders for the SAM of the new lateral branch or organ. The cellular composition of the new SAM reflects the proportional sampling of these sectors. Due to strong spatial constraints, precursors likely sample only one ASC via its clonal descendants, particularly if *m* is small. c) Under the cellular model shown in b) it is reasonable to assume a single ASC bottleneck model for shoot branching. The schematic shows how each branch is founded by a different ASC, although founder selection is probabilistic (see d.). d) Shown is the fate of a mutation arising in the bottom segment of the tree (see lighting bolt). How the mutation propagates to the crown tips depends on the sampling probabilities along the branching paths. Left panel: At branch point ℓ=0, the mutation is selected into a lateral branch with probability 1/m, where it becomes fixed in all subtrees descending from that branch point. At the same time, the mutant cell propagates further along the main stem, but—in this case—fails to be sampled at the terminal tip. Right panel: An alternative outcome, where the mutation fails to be selected into the lateral branch at ℓ=0, but propagates all the way to the tip of the main stem. The sampling probabilities of a mutation arising on any segment *s* to be observed in the tips of the crown are derived in the main text. Panel b was modified from Johannes (2025) and Chen et al. (2024).

Our goal is to model how somatic mutations accumulate across the tree architectures described above. Among all possible variants, those arising in the SAM are the most consequential, as all aerial cell lineages originate from this tissue. SAM-derived mutations can spread across large sectors of the plant body, especially when transmitted into axillary meristems (AMs) that establish new lateral branches or organs (Fig. 3). These mutations are more likely to reach high cellular frequencies, making them readily detectable by sequencing, and they carry greater potential for functional impact at the whole-tissue level as well as a higher likelihood of entering the germline. By contrast, mutations arising outside the SAM are typically confined to small, functionally local cell sectors. Because they rarely expand clonally or contribute to reproductive lineages, their chance of long-term fixation is negligible. As a result, such mutations are both difficult to detect with current sequencing technologies and of limited evolutionary significance.

#### Cellular model of shoot branching.

To understand how SAM-derived mutations spread throughout the plant, we must posit a cellular model of shoot branching that captures how SAM lineages contribute to lateral meristem formation. The SAM is a stratified, self-renewing structure composed of two tunica layers (L1, L2) and one corpus layer (L3) (Lyndon 1998). Each layer is maintained by a small number of apical stem cells (ASCs) (typically m=3−5 per layer in species such as *Arabidopsis*, tomato, or seagrass; Burian et al. 2016; Yu et al. 2024) arranged at the shoot apex (Fig. 3a). During internode growth, ASCs divide asymmetrically: one daughter retains its stem cell identity, while its sister is displaced into the periphery and contributes to organogenesis. This produces coherent clonal sectors within each layer, each tracing back to a single ASC (Fig. 3b).

Lateral branches arise from AMs, which form in the axils of developing leaves from small groups of SAM peripheral cells (Shi et al. 2016). Although multiple peripheral cells can initiate an AM, sampling is spatially restricted and often captures only a single ASC clonal sector per layer (Burian et al. 2016; He et al. 2024; Johannes 2025) (Fig. 3b,c). We therefore adopt a *single-ASC bottleneck model* at each branching event: one ASC (per layer) is probabilistically chosen (via its daughter cells at the SAM periphery) to found the new meristem.

We acknowledge that this branching model is a simplification as precursor sampling can span clonal boundaries and momentarily incorporate multiple ASC lineages, leading to polyclonal AMs (e.g. Bossinger and Smyth 1996; Burian et al. 2016). Indeed, Burian et al. (2016) estimate that roughly 39% of L1 AMs in Arabidopsis receive input from more than one ASC. However, subsequent branching events create additional bottlenecks that rapidly dilute mixed founder populations down to a single surviving lineage, as shown by Johannes (2025). Moreover, in the Appendix, we demonstrate that allowing any small number of ASCs to found each new meristem leads to the same effective propagation behavior as our single-cell model. Thus, the single-ASC bottleneck captures the essential dynamics of lineage fixation and somatic drift while retaining analytic simplicity. We now proceed to develop the mutation model, starting with a single SAM layer before extending to all three.

#### ASC sampling probabilities during branching.

Consider a mutation arising within the ASC population on segment *s* (Fig. 3d). Above this segment lie $B_{s}=D-s$ branch points where lateral shoots are formed. At each of these exactly one of the *m* ASC is randomly selected via its clonal descendants at the SAM periphery to act as a founder to a new lateral meristem (see Appendix for a relaxation of this assumption). The continuing axis itself terminates in a final branch point where terminal organs such as fruit or leaves are formed (Fig. 3d). Similar to lateral branching, we treat this terminal branch point as a cellular bottleneck, where one ASC founding cell is drawn uniformly from the *m* remaining cells. To be detected in a leaf or fruit, a mutation in *s* must therefore be selected in any one of the $B_{s}$ lateral-branch draws or in the terminal founder draw for a total of $B_{s}+1$ independent sampling events. Label these sampling events $j=1,2,\ldots ,B_{s}+1$ and let


$$Z_{j}=\left\{ \begin{array}{cc} 1, & \text{if the mutant cell is selected at event}\;j,\\ 0, & \text{otherwise}, \end{array}\right.$$

with each $Z_{j}∼\mathrm{Bernoulli}(1/m)$ (Fig. 3d). Using this formalism, it is useful to define two key probabilities:

1. Lateral-branch entry probability: The chance that the mutation enters a lateral branch at least once among the $B_{s}$ branch points (ignoring the final draw) is


$$q_{s}=1-\text{Pr}(Z_{1}=\cdots =Z_{B_{s}}=0)=1-{\left(1-\frac{1}{m}\right)}^{B_{s}}.$$

2. Unique-event detection probability: The chance that the mutation is selected in *any* of the $B_{s}+1$ draws (including the terminal founder) is


$$p_{\det}(s)=1-\text{Pr}(Z_{1}=\cdots =Z_{B_{s}+1}=0)=1-{\left(1-\frac{1}{m}\right)}^{B_{s}+1}.$$

Since the probability of failing all $B_{s}$ branch draws is $1-q_{s}$, one can write


$$p_{\det}(s)=q_{s}+(1-q_{s})\;\frac{1}{m}.$$

This decomposition partitions mutation detection into two disjoint events: “selection into a lateral branch” (probability $q_{s}$) or “no lateral selection but success at the terminal draw” (probability $(1-q_{s})/m$). These two shorthand probabilities, $q_{s}$ and $p_{\det}(s)$, capture the entire sampling dynamics and serve as the building blocks for all crown-wide mutation burden expectations described below.

### Expected somatic mutation burdens

#### Per-tip mutation burden.

In any internode segment *s* each of the *m* cells mutates according to a Poisson process with rate 2Gμ over time $t_{s}=T\;w_{s}(\lambda )$, so that the total mutational supply from the ASC population of single shoot is $m\;2G\mu \;t_{s}$. Only one of those *m* cells is eventually sampled at the terminal founder with probability 1/m so thinning yields $\left(2G\mu \;t_{s}\right)$. Summing over all segments from root to tip gives the expected per-tip mutation burden:


$$E[S_{\mathrm{tip}}]=\sum_{s=0}^{D}2G\mu \;t_{s}=2G\mu \;T.$$

In practical terms, this would be the expected number of somatic mutations observed in a single sequenced lateral organ (e.g. a leaf or fruit), given the total age of the tree (*T*), the haploid genome size (*G*) and the mutation rate (per-ASC per unit time).

#### Crown-wide mutation burden.

Let us now extend this reasoning to the entire crown by summing contributions from all internode segments. At level *s* of a perfectly balanced binary tree, there are $2^{s}$ internode segments, each harboring *m* ASCs that mutate as a Poisson process with rate 2Gμ over time $t_{s}=Tw_{s}(\lambda )$. The total mutational input from that level is therefore


$$2^{s}m(2G\mu t_{s}).$$

Mutations arising on a segment at level *s* must traverse $B_{s}=D-s$ branching events. At each branch point, exactly one of the *m* ASCs is chosen as founder, so the mutant lineage enters a new lateral branch with probability 1/m. These lateral branches form disjoint subtrees of sizes $2^{B_{s}-1},2^{B_{s}-2},\ldots ,2^{0}$, and the terminal branch contributes one additional tip. Summing these disjoint contributions gives the expected number of tips reached by such a mutation:


$$E[\text{tips}|s]=\sum_{\;j=1}^{B_{s}}\frac{1}{m}2^{B_{s}-j}+\frac{1}{m}=\frac{2^{B_{s}}}{m}.$$

By linearity of expectation, multiplying by the mutational supply and summing over all levels yields


$$E\left[S_{\mathrm{crown}}^{\mathrm{tips}}(\lambda )\right]=\sum_{s=0}^{D}2^{s}m(2G\mu t_{s})\frac{2^{D-s}}{m}=2G\mu 2^{D}T.$$

This result shows that the expected total mutation burden is proportional to the total number of terminal tips ($2^{D}$) multiplied by the developmental time along each root-to-tip path (*T*). In other words, it scales with the total integrated path length of the crown and is independent of the number of ASCs (*m*) and the way developmental time is distributed among segments ($w_{s}(\lambda )$).

#### Crown-wide unique-mutation burden.

Developmental time allocation within the crown becomes fundamentally important when we focus instead on the *number of unique somatic mutations* i.e. those counted once regardless of how many tips inherit them. In this case, we collapse the subtree-amplification factor $2^{B_{s}}$ into a single “seen-or-not” outcome that depends on the chance a mutation arising on segment *s* is transmitted through at least one of the $B_{s}$ lateral bottlenecks or through the terminal founder. Recall the probability that a mutation from level *s* is represented in at least one crown tip is


$$p_{\det}(s)=1-{\left(1-\frac{1}{m}\right)}^{B_{s}+1},\;B_{s}=D-s,$$

which increases for segments located deeper in the crown because they must pass through more branch points.

To obtain the expected number of unique mutations across the crown, we again sum by linearity of expectation over all levels to obtain:


$$E\left[S_{\mathrm{crown}}^{\mathrm{unique}}(\lambda )\right]=2G\mu mT\sum_{s=0}^{D}2^{s}w_{s}(\lambda )p_{\det}(s).$$

This expression defines the expected *unique* mutation burden in the crown as a function of architectural parameters. The architecture enters through two terms: (i) the developmental-time weights $w_{s}(\lambda )$, which describe how growth effort is distributed along the crown, and (ii) the detection probabilities $p_{\det}(s)$, which increase for segments with many downstream branch points. Together, these terms quantify how the spatial structure and temporal allocation of development jointly determine the number of distinct mutations that ultimately appear in the crown.

#### Remark on polyclonal founding.

Although we have built the above with a single-ASC bottleneck for clarity, empirical studies have found that AMs are occasionally founded by multiple stem cells (e.g. Bossinger and Smyth 1996; Burian et al. 2016; Johannes 2025). However, one can show (Appendix) that sampling any fixed number k≪m of ASCs at each branch point leads to the same *net* propagation probability 1/m per branch-and-fixation cycle. Thus, all of our tip- and crown-wide burden formulas—and their architectural dependence—remain approximately as stated.

#### Simulation of crown-wide mutation burden.

**Fig. 4.**
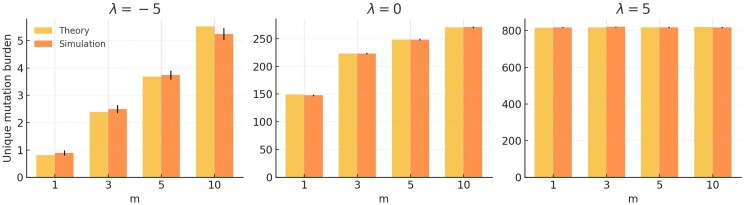
Simulation study. Evidence that the theoretical expectations agree with stochastic simulations. Example result from simulations of the crown-wide unique-mutation burden, $E[S_{\mathrm{crown}}^{\mathrm{unique}}(\lambda )]$, for D=10, m∈{1,3,5,10} and λ∈{−5,0,5}. Error bars are empirical 95% confidence intervals from 300 replicate runs. Full simulation results for a wider range of values can be found in Supplementary Table 1.

To validate the derived expectations, we implemented a Monte Carlo simulator that, for each combination of *D*, *m*, and *λ*, performs three steps: **1.** allocates segment times via the tilt function. **2.** draws Poisson-distributed mutational inputs in each segment for both tip-specific and crown-wide processes, and **3.** propagates each mutation through the $B_{s}$ lateral-branch and terminal founder bottlenecks exactly as in the model. We find that the simulation closely agree with the theoretical expectations (Fig. 4, Supplementary Table 1).

### Architectural amplification of unique somatic mutation burden

The results above show that while the total number of mutation *occurrences* across tips is fixed by the overall tree size and total developmental time, the number of *unique* somatic mutations is strongly shaped by branching architecture. Because the prefactor 2GμmT is constant for a given tree, we can write:


$$E\left[S_{\mathrm{crown}}^{\mathrm{unique}}(\lambda )\right]\propto A(\lambda ),$$

where


$$A(\lambda )=\sum_{s=0}^{D}2^{s}w_{s}(\lambda )\;p_{\det}(s)$$

captures the purely architectural contribution to the expectation.

#### Fold-change in unique-mutation burden between architectural extremes.

To quantify this architectural dependence, we examine two limiting growth regimes. When λ→−∞ (the *stick tree*), all developmental time is concentrated in the basal segment (s=0), giving


$$A(\mathrm{stick})=1-{\left(1-\frac{1}{m}\right)}^{D+1}.$$

Conversely, when λ→+∞ (the *star tree*), all time is devoted to the terminal segments (s=D), yielding


$$A(\mathrm{star})=\frac{2^{D}}{m}.$$

Because A(λ) is a convex combination of the segment-wise terms


$$f_{s}=2^{s}\left[1-{\left(1-\frac{1}{m}\right)}^{D-s+1}\right],$$

it satisfies


$$1-{\left(1-\frac{1}{m}\right)}^{D+1}\leq A(\lambda )\leq \frac{2^{D}}{m}.$$

The ratio between these extremes,


$$\frac{A(\mathrm{star})}{A(\mathrm{stick})}=\frac{2^{D}/m}{1-(1-1/m{)}^{D+1}},$$

illustrates the potential scale of architectural effects: this ratio can become very large, often several orders of magnitude, depending on the values of *D* and *m*.

#### Fold-change in unique-mutation burden relative to uniform allocation.

An alternative perspective is to compare each growth regime to one with uniform time allocation. When λ=0, developmental time is distributed evenly across all internode segments ($w_{s}=1/(D+1)$), defining a baseline A(0). We can then express the relative amplification or suppression of the unique-mutation burden as


$$R(\lambda )=\frac{A(\lambda )}{A(0)}.$$

**Fig. 5.**
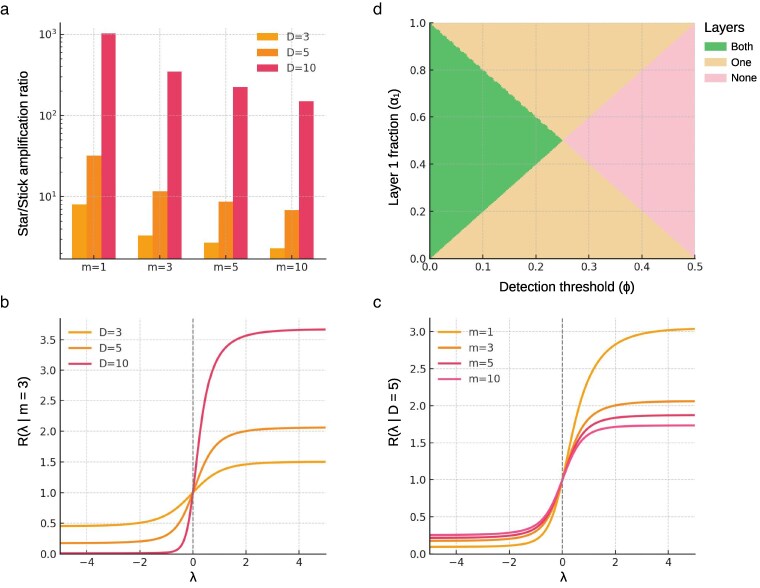
Architectural impact on somatic mutation burden. a) Fold-change differences in the unique-mutation burden between the star-tree architecture relative to the stick-tree architecture plotted on a *log* scale. The magnitude of the fold-change depends mainly on the depth *D* of the tree, but also on the ASC population size. b) For a fixed ASC (m=3), fold-change difference in the unique-mutation burden for different values of *λ* to a baseline architecture with uniform growth (λ=0). c) For a fixed depth (D=5), fold-change difference in the unique-mutation burden for different values of *λ* relative to a baseline architecture with uniform growth (λ=0). d) Impact of variant allele threshold (*x*-axis) in sequencing data of lateral organs (e.g. fruits) on the ability to observe mutations originating in any of the three meristematic layers. For visual simplicity, we assume that only two layers contribute cells to the sequenced sample. Depending on the threshold, mutations can be detected either from both layers (green), one layer (beige) or no layer (pink).

Here, R(0)=1, while R<1 indicates architectural suppression and R>1 indicates amplification relative to the uniform case. Even modest deviations from uniform growth (|λ|≈1) can shift the expected number of unique mutations by a factor of two to five, depending on *D* and *m* (Fig. 5b,c).

### Impact of cell-layer structure and detection thresholds

The preceding analysis has so far ignored the cellular organization of the SAM, which consists of three histological layers, L1, L2, and L3, each contributing to distinct tissue types (Lyndon 1998). When tissues such as leaves or fruits are sampled in bulk, these layers mix in unknown proportions $\alpha_{1}$, $\alpha_{2}$, and $\alpha_{3}$, satisfying


$$\sum_{k=1}^{3}\alpha_{k}=1.$$

Because the layers develop semi-independently, they may accumulate somatic mutations at different per-base rates $\mu_{k}$ (Goel et al. 2024), even though they share the same architectural parameters $\left\{B_{s},w_{s}(\lambda )\right\}$, genome size *G*, and stem-cell number *m*. The expected crown-wide number of *unique* somatic mutations in layer *k* can be written compactly as


$$E\left[S_{\mathrm{crown}}^{\mathrm{unique},(k)}\right]=2G\mu_{k}mT\;A(\lambda ),\;k=1,2,3.$$

Summing over all three layers gives


$$2GmT\;A(\lambda )\sum_{k=1}^{3}\mu_{k}.$$

In most empirical studies, bulk sequencing combines DNA from all three layers. Somatic mutations then appear as variant alleles at frequencies


$$f_{k}=\frac{\alpha_{k}}{2}$$

in diploid tissue. Because variant callers require a minimum allele-frequency threshold *ϕ* (typically 5% to 10%) to distinguish true mutations from sequencing error, a mutation from layer *k* is detected only if


$$f_{k}\geq \phi ⟺\alpha_{k}\geq 2\phi .$$

We represent this condition using the indicator function


$$\delta_{k}(\phi )=\left\{ \begin{array}{cc} 1, & \alpha_{k}\geq 2\phi ,\\ 0, & \alpha_{k}<2\phi . \end{array}\right.$$

Under this bulk-sequencing constraint, the expected number of detected *unique* somatic mutations becomes


(1) $$E\left[S_{\mathrm{crown}}^{\mathrm{unique},(\phi )}\right]=2GmT\;A(\lambda )\sum_{k=1}^{3}\delta_{k}(\phi )\mu_{k}.$$

The above equation shows that detection thresholds scale the expectation linearly by penalizing layers whose contribution $\alpha_{k}$ falls below the detection limit 2ϕ. Because $\sum_{k=1}^{3}\alpha_{k}=1$, the mixture automatically weights each layer’s mutation rate by its relative tissue fraction. In practice, layers with small $\alpha_{k}$ (for example, L3 in many species) may therefore become nearly invisible in bulk data, biasing empirical estimates of the crown-wide mutation burden toward the dominant layers (Fig. 5d). Although the effect of layer composition on the expectation was derived here for $E[S_{\mathrm{crown}}^{\mathrm{unique}}]$, the same logic applies directly to the total tip-level expectation $E[S_{\mathrm{crown}}^{\mathrm{tips}}]$.

## Conclusion

Somatic mutations arise as a natural consequence of cell division during development, and their accumulation in long-lived plants has broad implications for ecology, evolution, and genome stability. Surprisingly, genomic studies have consistently reported low somatic mutation counts in mature trees (Johannes 2024). Based on a rough calculation that did not take branching architecture into account, Schoen and Schultz (2019) argued that these counts should be several orders of magnitude higher, and attributed this discrepancy to errors in ascertaining mutations from sequencing data. While incomplete detection due to low allele frequencies in bulk sequencing may contribute, recent studies using layer-enriched or long-read calling approaches have yielded similar results (Goel et al. 2024; Johannes 2025; Xian et al. 2025), suggesting that technical artifacts are not the primary explanation.

Instead, we propose that the spatio-temporal structure of the tree crown itself strongly suppress the accumulation of fixed somatic mutations. Because branching events are accompanied by tight cellular bottlenecks, and because terminal branches often share long portions of their developmental history, many cell divisions do not generate new, independently traceable mutations. Instead, the degree of developmental path-sharing among branches plays a key role in determining the likelihood that a mutation arising in a stem cell lineage becomes fixed and observable in the crown.

This same logic applies to somatic epimutations, which typically occur at much higher rates than DNA sequence mutations (Becker et al. 2011; Schmitz et al. 2011; van der Graaf et al. 2015; Hazarika et al. 2022; Yao et al. 2023), but exhibit similar clonal inheritance in meristematic tissues (Hofmeister et al. 2020; Shahryary et al. 2020). Although epimutations may revert over long timescales (van der Graaf et al. 2015), such reversions are rare over the lifespan of a tree. Thus, our modeling framework extends naturally to both types of heritable molecular change, requiring no reparameterization.

Our model generates several empirically testable predictions. For example, trees with more path-sharing among terminal branches, those in which branching occurs late in development, are expected to exhibit a lower burden of unique somatic mutations than trees in which branches diverge early and grow independently. As a result, mutation accumulation should vary with branching strategy even when crown depth and number of tips are held constant. Moreover, because each branching event samples from a limited set of stem cell lineages, mutation accumulation should saturate with developmental depth. That is, adding more branching events beyond a certain point contributes little additional diversity, as the number of distinct ASC lineages that can be sampled is limited. This saturation effect implies that mutation counts should increase sublinearly with path length. Within a single tree, mutation sharing between terminal branches should reflect their developmental proximity: branches that diverged more recently should share more mutations than those that separated earlier. These predictions are amenable to testing through phylogenetic analysis of branches or direct quantification of mutation burden across trees with contrasting crown architectures. Furthermore, they suggest that experimentally altering branching patterns—for instance, through pruning or hormone treatments—should modulate mutation accumulation in predictable ways.

Our work complements very recent efforts by Tomimoto et al. (2025) who examined how the mean genetic difference between all pairs of branches of a tree varies as a function of the ratio between main-lateral or daughter-mother shoots. A caveat with both analytical approaches is that they focus exclusively on mutations arising in the ASC within the SAM. This emphasis reflects the fact that ASC-derived mutations are more likely to become fixed due to the cellular bottlenecks at branching events, occur at high enough cellular frequencies to be reliably detected through genomic sequencing, are more amenable to mathematical modeling, and are likely to be functionally significant. By contrast, mutations that arise post-SAM, during organogenesis or later development, are expected to occupy small, localized sectors. Their distribution is less predictable and more difficult to model in relation to the overall tree architecture. Nevertheless, because such mutations do occur, our model offers a lower bound on the cumulative burden of somatic mutations in trees.

Taken together, our theoretical insights emphasize that tree architecture is not merely a consequence of growth, but also a developmental scaffold that governs the accumulation and distribution of somatic (epi)mutations. By linking growth form with molecular evolution, this framework opens new directions for studying how life-history strategies shape genetic and epigenetic diversity in long-lived plants.

## Supplementary Material

jkaf234_Supplementary_Data

## Data Availability

No empirical data generated. Supplemental material available at G3 online.

## Appendix: Robustness of the architectural factor to multi-ASC sampling

In the main text, we assumed that each branching event is founded by a single ASC per layer. This yields a per-branch propagation (or “branch-and-fix”) probability of 1/m for a mutation that is present in exactly one of the *m* ASCs on a segment. Here, we show that allowing a small number b≪m of ASCs to found each new meristem leaves the architectural dependence of the expected crown-wide unique-mutation burden essentially unchanged.

### Setup and notation

We keep the tree-level notation from the main text:

- *m*: number of ASCs per SAM layer.
- *b*: number of ASCs sampled *without replacement* at each branch (lateral) or terminal event, with 1≤b≪m.
- *D*: crown depth; segments indexed by s=0,1,…,D.
- $B_{s}=D-s$ : number of branch points above segment *s*.
- $2^{s}$ : number of internode segments at level *s*.
- $T,G,\mu ,w_{s}(\lambda )$ : total developmental time, haploid genome size, per-base mutation rate, and time-allocation weights, respectively.

Under the single-ASC model, the expected crown-wide number of unique somatic mutations was

$$E\left[S_{\mathrm{crown}}^{\mathrm{unique}}(\lambda )\right]=2G\mu mTA(\lambda ),$$

with the architectural factor

$$A(\lambda )=\sum_{s=0}^{D}2^{s}w_{s}(\lambda )\left[1-{\left(1-\frac{1}{m}\right)}^{B_{s}+1}\right].$$

We now re-derive the same quantity when b>1 founders are sampled at each bottleneck.

### Seeding probability when *b* founders are drawn

Suppose a mutation is present in exactly one of the *m* ASCs on segment *s*. At the next branching event we draw *b* ASCs without replacement to found the daughter meristem. Biologically, this “seeding” step corresponds to peripheral SAM descendants that are recruited to form the new AM. The probability that at least one of these *b* founders is the mutant is

rb=1−(m−1b)(mb)=bm.

Immediately after seeding, the new meristem contains *b* founder lineages, one of which carries the mutation, each at frequency 1/b.

### Effective branch-and-fix probability for non-terminal levels

For segments s<D, each branching event involves two steps:

1. **Seeding:** draw *b* founders from the parent SAM; this captures the mutant with probability $r_{b}=b/m$.
2. **Fixation:** at the next bottleneck along that lineage, only one of the *b* founder lineages will be continued (or will dominate) in the descendant axis; this happens with probability 1/b for the mutant lineage.

Biologically, the second step represents the fact that descendant branches quickly pass through another narrow sampling event, so mixed founders do not persist indefinitely. The net probability that the mutation survives a full branch-and-fix cycle is therefore

$$r_{b}\times \frac{1}{b}=\frac{b}{m}\times \frac{1}{b}=\frac{1}{m}.$$

Hence, for all non-terminal segments, the propagation probability per branching event remains 1/m, identical to the single-ASC model.

### Terminal level (s=D)

Mutations arising on the terminal segment have no further lateral branches, only the final draw of *b* ASCs. In this case, the detection probability simplifies to

$$p_{\det}^{\left(b\right)}(D)=\frac{b}{m},$$

instead of 1/m.

### Architectural factor under multi-ASC founding

Mutations that arise on segments s=0,1,…,D−1 still face $B_{s}+1=D-s+1$ independent branch-and-fix events, each succeeding with probability 1/m, giving

$$p_{\det}^{\left(b\right)}(s)=1-{\left(1-\frac{1}{m}\right)}^{B_{s}+1},\;s=0,1,\ldots ,D-1.$$

Only the terminal-level term changes to b/m. The modified architectural factor therefore becomes

$$A_{b}(\lambda )=\sum_{s=0}^{D-1}2^{s}w_{s}(\lambda )\left[1-{\left(1-\frac{1}{m}\right)}^{B_{s}+1}\right]+2^{D}\;w_{D}(\lambda )\;\frac{b}{m}.$$

The corresponding crown-wide expectation is

$$E\left[S_{\mathrm{crown}}^{\mathrm{unique}}(\lambda )\right]=2G\mu mTA_{b}(\lambda ).$$

### Practical impact

In most realistic architectures, the time weight on the terminal level, $w_{D}(\lambda )$, is small, and b≪m. In this regime the correction term in $A_{b}(\lambda )$ is negligible, so

$$A_{b}(\lambda )\approx A(\lambda ),$$

and all qualitative conclusions in the main text about architectural amplification or suppression of the crown-wide unique mutation burden continue to hold.
